# Effect of Root Canal Filling Materials and Pretreatment with Solvents on the Shear Bond Strength of Composite Resin with Primary Tooth Dentin

**DOI:** 10.1155/2021/5534294

**Published:** 2021-04-02

**Authors:** Taraneh Zeynalzade Ghouchani, Hajar Farhadpour, Najmeh Mohammadi

**Affiliations:** ^1^Department of Pediatric Dentistry, School of Dentistry, Shiraz University of Medical Sciences, Shiraz, Iran; ^2^Department of Operative Dentistry, School of Dentistry, Shiraz University of Medical Sciences, Shiraz, Iran

## Abstract

**Aim:**

Root canal filling materials have the tendency to inhibit adhesion of resin-based composites. This study was aimed at evaluating the effect of root canal filling materials and their solvents on the shear bond strength (SBS) of resin composite with the primary tooth dentin.

**Methods and Materials:**

Seventy-two intact anterior primary teeth were selected. Smooth dentinal surfaces were prepared to a minimum diameter of 3 mm and thickness of 1.5–2.0 mm. The samples were equally divided into six groups (*n* = 12). In group 1: control group, no root filling material; in group 2: Metapex, no solvent; in group 3: Metapex+ethanol solvent; in group 4: ZOE, no solvent; in group 5: ZOE+ethanol solvent; and in group 6: ZOE+orange oil solvent were applied. Then, dentin surfaces were etched, and composite restorations were placed and cured. The specimens were stored in distilled water at 37°C for 24 hours. SBS values were determined using a universal testing machine.

**Results:**

The SBS values of composite to dentin in groups 2 and 4 were significantly lower than those in the control group (*P* < 0.001). Cleansing of the specimens with 96% ethanol after removal of Metapex significantly increased the composite-dentin bond (*P* < 0.001). Applying ZOE, only orange oil solvent significantly increased the SBS of the composite to the primary tooth dentin (*P* = 0.01).

**Conclusion:**

To reduce the negative effects of endodontic root filling materials on the SBS of composite and primary tooth dentin, ethanol is a suitable solvent when Metapex is used, while orange oil might be a better choice than ethanol when applying ZOE.

## 1. Introduction

Nowadays, the use of composites is on the rise in pediatric restorative dentistry [1]. These materials directly bond with the tooth structure by reinforcing it. This property is beneficial for endodontically treated teeth, which usually require extensive restorations [2].

Due to the complex chemical composition of the resin, bond strength is affected by various factors such as contaminants like blood, saliva, handpiece lubricants, liners, bases, and intracanal filling materials [3]. In the 1930s, zinc oxide eugenol (ZOE) paste was introduced as the first root canal filling material for primary teeth [4], making it the most commonly used filling material for pulpectomy of primary teeth.

However, nowadays, other novel root filling materials are being used for pulp therapy of primary teeth. Several studies were published in Japan on calcium hydroxide (Ca(OH)_2_) and iodoform mixture. Based on the results of the study by McTigue et al. [5], Ca(OH)_2_/iodoform mixture meets the criteria of an ideal primary tooth filling material. Use of an iodoform-based paste or a material containing Ca(OH)_2_, as a substitute for ZOE, has gained attention in recent years. These materials are easy to apply, resorb at a slightly faster rate than the physiologic resorption of primary teeth roots, have no toxic effect on the permanent successor, and are radiopaque. For these reasons, Ca(OH)_2_/iodoform mixture is considered to be nearly an ideal primary tooth root filling material [6].

Root canal filling materials have tendency to inhibit adhesion between the resin-based composite materials and dentin surface [7]. Although some studies have concluded that eugenol-containing sealers do not have an inhibitory effect on the bond strength of resin based materials, there is still disagreement regarding this issue [8].

ZOE might affect bonding in two ways: first, the residual material in dentinal tubules might affect the bonding process. Second, hydrolysis of hardened material might occur as the result of exposure to water. Release of eugenol prevents polymerization of resin-based materials [9].

In addition to ZOE, effect of other root canal filling materials containing Ca(OH)_2_/iodoform on bond strength of resin-based materials to primary tooth dentin was evaluated in recent studies. It was reported that calcium hydroxide does not affect bonding of resin-based materials [10]. However, there are limited reports concerning the effect of iodoform on the bond strength of resin-based materials. Iodoform is slightly dissolved in water. This material is very soluble in ethanol, acetone, ether, and benzene [11].

In some studies, it was shown that Metapex (calcium hydroxide/iodoform mixture) results in reducing the bonding of adhesives to primary tooth dentin. There are two scenarios; one is that remnant material directly reduces the bonding ability (as it was told for ZOE), or remnant Metapex might be dissolved when exposed to adhesive primer which contains ethanol or acetone, hence, reducing the bond strength [12].

In order to minimize the effect of root filling materials on bond strength of resin-based materials, several solutions have been proposed [13]. The most common method is pretreatment of dentin with different chemical solvents [14]. Based on the results of recent studies, ethanol is suggested as the most common solvent when Metapex or ZOE are used as root filling materials. This solvent is effective in reducing the adverse effects of root filling materials on bonding to primary tooth dentin [15].

Essential oils extracted from the peel of sweet orange, Citrus aurantium, are an excellent alternative organic solvent compared to potentially toxic solvents, especially used on zinc oxide eugenol-based cements [16]. Orange oil is a lanoline-based essential oil, the effects of which have been evaluated on retentive strength of metallic cast restorations cemented temporarily with zinc oxide eugenol [17]. In a recent study, this novel material was recommended as a biocompatible product for endodontic retreatment procedures [18].

However, to the best of the authors' knowledge, no research has evaluated this material as a solvent after root canal filling of primary teeth with ZOE. The purpose of this study was to evaluate the effect of root canal filling materials (ZOE and Metapex), besides pretreatment with the aforementioned solvents on the shear bond strength (SBS) of composite resin with primary tooth dentin.

## 2. Methods and Materials

After obtaining written informed consent from the parents or guardians, seventy-two extracted intact human anterior primary teeth (extracted for orthodontic reasons) were used in this study. Freshly extracted teeth (within 3 months) were disinfected in 0.5% chloramine T and stored in distilled water at 37°C. The roots were removed perpendicular to the long axis of the teeth with a 0.3 mm thick diamond blade (Minitom, Struers A/S, Copenhagen, Denmark); and the crowns were cut in mesiodistal direction to expose pulp chamber dentin. Then, the samples were embedded in chemically cured acrylic resin (Acropars, Kaveh, Tehran, Iran) as their dentinal surface was exposed. A smooth dentin surface was prepared to a minimum diameter of 3 mm and thickness of 1.5–2.0 mm by using a fissure bur (Tizkavan, Tehran, Iran), on a high-speed handpiece (NSK, Japan) under water and air spray. Specimens were lapped manually with wet 600-grit silicon carbide paper (Struers A/S, Copenhagen, Denmark) to produce a flat surface.

Dentin specimens were equally divided into six groups (*n* = 12):

*Group 1*: control: no root canal filling material was used. Dentin was cleaned by a cotton pellet with normal saline.

*Group 2*: Metapex (Meta Biomed Co. Ltd, Cheongju, Korea) was applied to the dentin and rubbed with a cotton swab to simulate the impregnation of the dentin with root filling materials during pulpectomy procedure. Consecutively, the remnant sealer on the dentin was cleaned with an excavator and a cotton pellet with normal saline.

*Group 3*: the same steps as group 2 were done, but the remnant sealer on the dentin was cleaned with an excavator and a cotton pellet with ethanol. Ethanol was applied for approximately 1 min until the surface appeared visibly clean. Then, the surface was cleaned with a cotton pellet with normal saline.

*Group 4*: ZOE (Kemdent Work Ltd, England) was applied to the dentin and rubbed with a cotton swab. Consecutively, the remnant sealer was cleaned with an excavator and a cotton pellet with normal saline.

*Group 5*: the same steps as group 4 were done, but the remnant sealer on the dentin was cleaned with an excavator and a cotton pellet with ethanol. Ethanol was applied for approximately 1 min until the surface appeared visibly clean. Then, the surface was cleaned with a cotton pellet with normal saline.

*Group 6*: the same steps as group 4 were done, but the remnant sealer was cleaned with an excavator and a cotton pellet with orange oil (Nipponshika, Yakuhin Co. Ltd, Shimonoseki, Japan). One to 2 drops of orange oil were applied on the dentin and allowed to act during 5 min (based on the proposed protocol). Then, the surface was cleaned with a cotton pellet with normal saline.

After treating the dentin surfaces with the aforementioned protocols, two samples from each group were randomly separated and examined by a scanning electron microscope (SEM) for visualization of the openings of dentinal tubules before restoration placement. The remaining 10 dentin samples in each group were etched with 35% phosphoric acid (Scotchbond Etchant, 3M ESPE) for 15 s and rinsed with water for 10 s, then gently dried with cotton pellets and air spray. Further, two layers of adhesive (3M Bond, USA) were applied with a microbrush, and each layer was dispersed by applying a weak air stream and then cured with a light emitting diode curing unit (Bluephase C5, Ivoclar Vivadent clinical, Austria) for 20 s. A Clear plastic mold (Tygon tubes, ET, Shandong China) with an internal diameter of 2 mm and height of 2 mm was filled with composite (3M ESPE, USA) and placed on the dentin surface. The excess material was removed by a scalpel (Juya, Iran). Light curing was performed from four dimensions (one at the center and three lateral spots) with total time of 40 s. Clear plastic molds were then gently cut by a scalpel and separated from the composite. The description of materials used in this study is listed in Table 1.

The specimens were stored in distilled water at 37°C for 24 h; then, shear bond strength (SBS) values were determined using a universal testing machine (Zwick/Roll Z020, Zwick GmbH &Co, Ulm-Einsingen, Germany). The test was performed by securing the specimen in a mounting jig, and a sharp straightedge chisel attached to the crosshead was used to apply a shearing force of 0.5 mm/min until failure. By dividing the load in *N* by the internal surface area of the tubes (2 mm diameter), shear bond strength in megapascal was calculated.

### 2.1. Preparation for Visualization by Using Field-Emission Scanning Microscope (SEM)

Two samples from each group after treatment of dentin surface, according to the instructions for sample preparation in each group of the study, and before restoration were examined by SEM to observe root filling material penetration into dentinal tubules. Moreover, cut sections of sheared dentinal surfaces of each group were examined using magnifications of up to 1000 for analysis of surface morphology, with emphasis on areas of adhesive failure or areas of cohesive failure. The specimens were mounted on aluminum stubs with conductive silver liquid, gold sputtercoated and examined under a field-emission SEM (TE-SCAN, MIRA3, USA) to verify types of failure fracture.

### 2.2. Types of Failure Fracture

Types of failure fracture were examined under SEM. Failures were categorized as follows: adhesive failure at the dentin and adhesive interface, cohesive failure in composite, cohesive failure in dentin, or a mixed failure that points to partial cohesive and adhesive failure.

### 2.3. Statistical Analysis

The collected data were analyzed by using SPSS, version. 20 (SPSS Inc., IL, USA). Data were analyzed using one-way ANOVA and post hoc Tukey's tests. *P* values < 0.05 were considered to be statistically significant.

## 3. Results

The means SBS, standard deviation (SD), and minimum and maximum bond strengths (MPa) for all groups are presented in Table 2. Based on the results, the highest SBS value belonged to the control group (saline) (9.19 MPa). The lowest SBS belonged to the zinc oxide eugenol group with no solvent (5.50 MPa).

Data analysis with one-way ANOVA showed statistically significant difference between the groups (*P* < 0.001). Hence, post hoc analysis with Tukey test was done for pairwise comparison (Table 2).

Compared to the control group, impregnation of specimens with Metapex and ZOE significantly reduced the composite bond strength with the dentin (*P* < 0.001).

Cleansing the specimens with 96% ethanol after removal of Metapex in group 3 significantly increased the shear bond strength of composite with the dentin in comparison to the group 2 (*P* < 0.001). The SBS of this group (Metapex with ethanol solvent) and the control group were not statistically different (*P* = 0.62).

Cleansing of specimens with 96% ethanol after removal of ZOE in group 5 did not increase the SBS significantly in comparison to group 4 (*P* = 0.57). However, treatment of the specimens with orange oil solution in group 6 exhibited significantly higher SBS values than group 4 (*P* = 0.01).

After impregnation of specimens with ZOE, despite the application of solvents in groups 5 and 6 (ethanol 96% and orange oil), the mean SBS values remained significantly lower than the control group (*P* values = <0.001 and 0.01, respectively).

### 3.1. SEM Results

**Figure 1:**
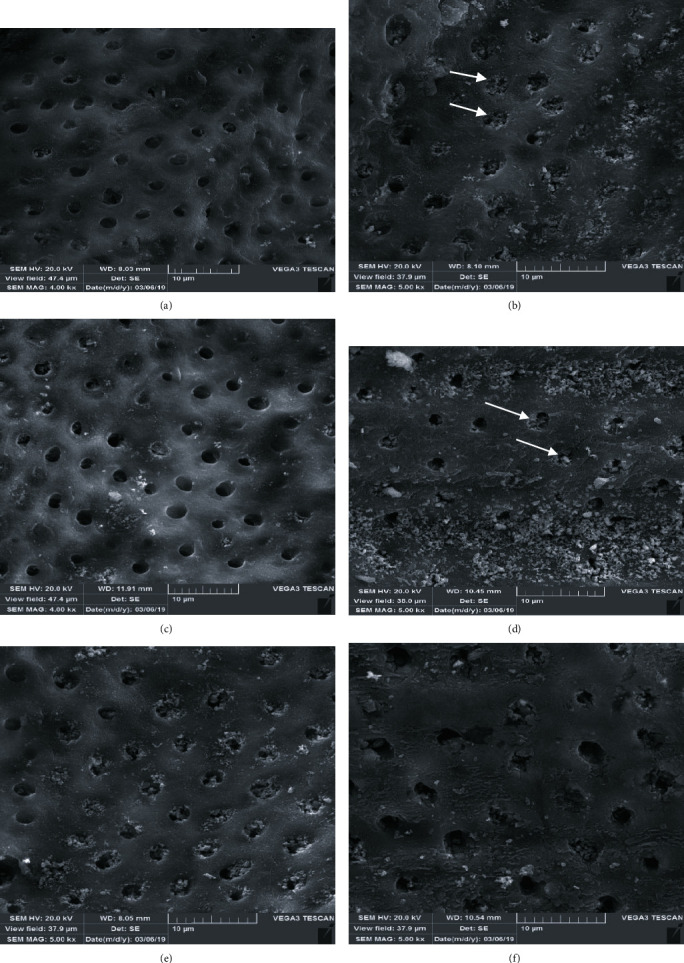
Representative images of dentin surfaces after different treatment protocols related to each group of the study: (a) group 1: control group, no filling material; (b) group 2: Metapex; (c) group 3: Metapex+ethanol solvent; (d) group 4: ZOE; (e) group 5: ZOE+ethanol solvent; (f) group 6: ZOE+orange oil solvent. Arrows show the orifice of dentinal tubules.

SEM images of dentin surfaces after different treatment protocols related to each group of the study are shown in Figure 1. As it is evident, SEM results confirm the findings of our study. Root filling materials of primary teeth block the dentinal tubules and result in decreasing the SBS of composite with the dentin, but cleansing the dentin surface with solvents was shown to be effective in reducing this negative effect.

**Figure 2:**
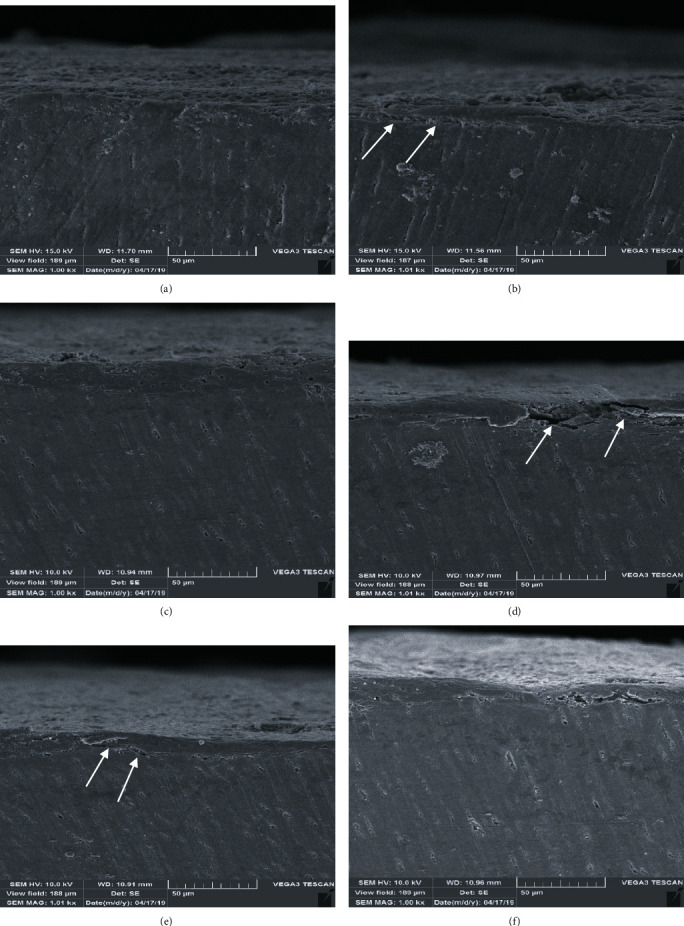
Representative images from visualization of cut sections of sheared dentinal surfaces by SEM: (a) group 1: control group, no filling material, no solvent; (b) group 2: Metapex; (c) group 3: Metapex+ethanol solvent; (d) group 4: ZOE; (e) group 5: ZOE+ethanol solvent; (f) group 6: ZOE+orange oil solvent. Arrows show the gap between the dentin and composite resin.

Figure 2 depicts representative images from visualization of cut sections from sheared dentinal surfaces by SEM that is also consistent with the mentioned data analysis. In images (b) (Metapex group) and (d) (ZOE group) that are related to the groups with the lowest SBS, presence of a gap and adhesive failure at the dentin and adhesive interface is evident.

## 4. Discussion

The purpose of this study was to evaluate the effect of root canal filling materials and their solvents on shear bond strength of composite with the dentin in the anterior primary teeth. Most of the composite resin restoration failures in anterior teeth are due to high shear forces [19]. Hence, we evaluated the effect of different root canal filling materials and their solvents on shear bond strength of composite to anterior primary teeth. Although it was reported that root canal sealers affect bond strength of resin-based materials to pulp chamber dentin in permanent teeth [12], less attention has been given to this subject in primary dentition.

The organic content, fluid-filled tubules, and variations in intrinsic composition of dentin make the bonding with dentin a greater challenge, compared with enamel [20]. Since pulp chamber dentin has structural and compositional differences from coronal dentin, it becomes a challenge for the adhesion of the dentin with the composites [21].

As it is known, diameters of dentinal tubules are larger in pulp chamber dentin than coronal dentin. Hence, the amount of intertubular dentin that has a strong source of collagen decreases from coronal to pulp chamber dentin. In this area, resin tags of hybrid layer cannot be properly attached to the dentinal walls [22]. Low adhesion values reported in this study can be due to the structural complexity in pulp chamber dentin, which led to lower bonding ability of resin-based materials.

Based on our results, Metapex and ZOE sealers significantly reduced the composite bond strength with the dentin. The same results were reported in other studies. Elbay and Tosun [12] in their study found that remnant of sealers in dentinal tubules can lead to reduced bonding. However, researchers have shared different opinions in this regard. This controversy can be related to variation in materials and testing methods [8, 23]. According to the reports of Peutzfeld and Asmussen [24] and Leirskar and Nordbø [23], ZOE has no negative effect on bond strength of adhesive systems. Carvalho et al. [25] also reported that ZOE has no negative impact on bonding strength of total etch systems, but it reduced bonding adhesion of self-etch systems. Elimination of negative effects of eugenol on total etch bonding groups could be due to acid etching and rinsing procedures [23].

Based on the results of the present study, the greatest decrease in SBS values was observed in the ZOE group. In prior studies, also, it was found that ZOE can reduce the bond strength of composite resin with dentin in two major ways: first, the remnant sealer in dentinal tubules might reduce the bonding adhesion. Second, after the process of hardening, water exposure can result in eugenol release [9]. Because of the existence of the hydroxyl group in the chemical structure of eugenol, polymerization of composite resin materials would be hindered [19]. Reduced bond strength values observed in this research can be the consequence of the aforementioned reasons, as it is confirmed in SEM images.

Metapex as a substation for ZOE has received great attention in recent years. In the present study, the effect of Metapex on the shear bond strength of composite with primary tooth dentin was also investigated. Major compounds of Metapex are calcium hydroxide and iodoform [5]. Various studies have shown that calcium hydroxide has no impact on the bond strength of composite resin with the dentin [10]. But there is no report about iodoform concerning this subject.

Our results showed that Metapex weakens the bonding adhesion. This reduction could be attributed to two main reasons that were also mentioned in other studies: remnant Metapex that directly affects the bonding and also dissolving of remnant Metapex material when exposed to adhesive primer, which contains ethanol and acetone [12].

A clean dentinal surface is necessary for proper bonding [21]. Most studies about surface treatment techniques to improve the adhesion are limited to permanent teeth [19]. But, in the present study, the effect of different solvents on the shear bond strength of composite with dentin in primary teeth was evaluated. Other researches have suggested ethanol as the most common chemical solvent before composite restoration, when Metapex or ZOE are used as root filling materials [15].

In this study, cleansing specimens with 96% ethanol after removal of Metapex in group 3 significantly increased the shear bond strength in comparison with group 2. The higher shear bond strength observed in this group could be due to dissolving effect of ethanol on remnants of Metapex in dentinal tubules.

Regarding ZOE and eugenol remnants, based on our results, 96% ethanol did not show significant effect on the SBS values. However, application of orange oil solvent (group 6) exhibited significantly better adhesion values in comparison with group 4 (ZOE with no solvent). Due to safety, noncarcinogenicity, and biocompatibility of orange oil, its application in endodontics is on the rise [16, 26].

To the best of our knowledge, no research has been conducted on the effect of this solvent on the shear bond strength of composite resin with dentin after root filling of primary teeth with ZOE.

According to the previous studies that introduced the orange oil as a specific solvent for zinc oxide eugenol-based cements and low dissolving efficacy of essential oil solvents on calcium hydroxide-based sealers observed in those studies [16, 27], the orange oil was not considered the solvent for Metapex sealer group in the present study.

The results of the representative images from visualization of sheared dentinal surfaces by SEM were also consistent with the mentioned data. In groups with the lowest SBS (groups 2 and 4), presence of gap and adhesive failure at the dentin and adhesive interface was evident. On the contrary, the most prevalent mode of failure in groups 3, 5, and 6 was mixed (cohesive and adhesive) failure type (Figure 2).

The results of this study should be confirmed by future clinical studies with larger sample sizes to evaluate techniques or materials.

## 5. Conclusion

To reduce the negative effects of endodontic root filling materials on the SBS of composite and primary tooth dentin, ethanol is a suitable solvent when Metapex is used, while orange oil might be a better choice than ethanol when applying ZOE.

## Figures and Tables

**Table 1:** Description of materials.

Material	Manufacturer	Description
Diamond blade	Minitom, Struers A/S, Copenhagen, Denmark	0.3 mm thick diamond blade
Fissure bur	Tizkavan, Tehran, Iran	
Acrylic resin	Acropars, Kaveh, Tehran, Iran	Chemically cured acrylic resin
Silicon carbide paper	Struers A/S, Copenhagen, Denmark	
Metapex	Meta Biomed Co. Ltd, Cheongju, Korea	Combination paste of iodoform and calcium hydroxide
ZOE	Kemdent Work Ltd, England	Zinc oxide eugenol containing root canal filling material
Ethanol		70% ethanol
Orange oil	Nipponshika, Yakuhin Co. Ltd, Shimonoseki, Japan	Essential oil, an extract of the peel of sweet orange fruit
Etchant	Scotchbond, 3M ESPE, USA	37% phosphoric acid gel
Bonding	Adper, Single Bond 2, 3M ESPE, USA	Adhesive
Universal restorative composite	Filtek Z250 XT, 3M ESPE, USA	Nanohybrid universal restorative composite, A_3_ shade

**Table 2:** Mean shear bond strength (MPa) and standard deviation for all the study groups.

Study groups	Number	Mean (MPa) ± SD	Minimum	Maximum	*P* value
Group 1: control^a^	10	9.19 ± 1.16	7.32	11.52	<0.001
Group 2: Metapex^cd^	10	5.74 ± 1.54	3.22	8.34	
Group 3: Metapex+ethanol^ab^	10	8.30 ± 0.49	7.79	9.14	
Group 4: ZOE^d^	10	5.50 ± 1.30	3.66	7.21	
Group 5: ZOE+ethanol^cd^	10	6.42 ± 1.13	5.01	8.49	
Group 6: ZOE+orange oil^bc^	10	7.30 ± 1.00	5.24	8.44	

At least one the same lowercase letter indicates lack of statistical difference (Tukey post hoc test).

## Data Availability

The authors will provide their data on request.

## Additional Points

*Clinical Significance*. Pretreatment of dentin with chemical solvents could be used as an effective treatment for improving the bonding performance and longevity of resin-based composite restorations in primary dentition.
